# Visualizing Patterns and Trends of 25 Years of Published Health Literacy Research

**DOI:** 10.3928/24748307-20170829-01

**Published:** 2017-10-10

**Authors:** Philip M. Massey, Meen Chul Kim, Prudence W. Dalrymple, Michelle L. Rogers, Kisha H. Hawthorne, Jennifer A. Manganello

## Abstract

**Background:**

With an increase in the number of disciplines contributing to health literacy scholarship, we sought to explore the nature of interdisciplinary research in the field.

**Objective:**

This study sought to describe disciplines that contribute to health literacy research and to quantify how disciplines draw from and contribute to an interdisciplinary evidence base, as measured by citation networks.

**Methods:**

We conducted a literature search for health literacy articles published between 1991 and 2015 in four bibliographic databases, producing 6,229 unique bibliographic records. We employed a scientometric tool (CiteSpace [Version 4.4.R1]) to quantify patterns in published health literacy research, including a visual path from cited discipline domains to citing discipline domains.

**Key Results:**

The number of health literacy publications increased each year between 1991 and 2015. Two spikes, in 2008 and 2013, correspond to the introduction of additional subject categories, including information science and communication. Two journals have been cited more than 2,000 times—the *Journal of General Internal Medicine* (*n* = 2,432) and *Patient Education and Counseling* (*n* = 2,252). The most recently cited journal added to the top 10 list of cited journals is the *Journal of Health Communication* (*n* = 989). Three main citation paths exist in the health literacy data set. Articles from the domain “medicine, medical, clinical” heavily cite from one domain (health, nursing, medicine), whereas articles from the domain “psychology, education, health” cite from two separate domains (health, nursing, medicine and psychology, education, social).

**Conclusions:**

Recent spikes in the number of published health literacy articles have been spurred by a greater diversity of disciplines contributing to the evidence base. However, despite the diversity of disciplines, citation paths indicate the presence of a few, self-contained disciplines contributing to most of the literature, suggesting a lack of interdisciplinary research. To address complex and evolving challenges in the health literacy field, interdisciplinary team science, that is, integrating science from across multiple disciplines, should continue to grow. **[*Health Literacy Research and Practice*. 2017;1(4):e182–e191.]**

**Plain Language Summary:**

The addition of diverse disciplines conducting health literacy scholarship has spurred recent spikes in the number of publications. However, citation paths suggest that interdisciplinary research can be strengthened. Findings directly align with the increasing emphasis on team science, and support opportunities and resources that incentivize interdisciplinary health literacy research.

The study of health literacy has significantly expanded over the past decade. It represents a dynamic area of inquiry that extends to multiple disciplines. Health literacy emerged as a derivative of literacy and early definitions focused on the ability to read and understand medical instructions and health care information (Parker, Baker, Williams, & Nurss, 1995; Williams et al., 1995). This early work led to a body of research demonstrating that people with low health literacy generally had poorer health outcomes, including lower levels of screening and medication adherence rates (Baker, Parker, Williams, Clark, & Nurss, 1997; DeWalt, Berkman, Sheridan, Lohr, & Pignone, 2004; Gazmararian et al., 2006).

Over time, the definition of health literacy expanded to include the capacity to obtain, process, understand, and apply health information and services required to make informed decisions that allow health-enhancing actions at the individual, social, and environmental levels (Institute of Medicine [US] Committee on Health Literacy, 2004; Nutbeam, 2008). This broader definition included skills and competencies that can be used to navigate through a complex health care system as well as in the broader health information environment outside of the clinical context (Jordan, Buchbinder, & Osborne, 2010; Lee, Arozullah, & Cho, 2004; Zarcadoolas, Pleasant, & Greer, 2005). In fact, most health literacy scholars now take the view that health literacy is a combination of individual literacy skills and the demands of the health care system (Parker & Ratzan, 2010).

Along with this expanding definition came a focus on health literacy by researchers from an increasing number of fields including medicine (Peterson, Shetterly, & Clarke, 2011; Williams et al., 1995), public health (Nutbeam, 2000; Sentell, Zhang, Davis, & Baker, 2014), education (Kilgour, Matthews, Christian, & Shire, 2015; Paakkari & Paakkari, 2012), nursing (Shieh & Belcher, 2013; Zanchetta, Taher, Fredericks, & Waddell, 2013), and information and library sciences (Dalrymple, Zach, & Rogers, 2014; Lawless, Toronto, & Grammatica, 2016). The multidisciplinary approach to the study of health literacy aligns with the National Research Council (2015) report, underscoring the importance of team science, or “research conducted by more than one individual in an interdependent fashion” as a way to address increasingly complex scientific and social challenges. The report goes on to say that team science is best accomplished through a diversity of interdisciplinary fields.

Drawing from the multiple definitions, conceptual dimensions, and disciplines of health literacy scholarship, Sørensen et al. (2012) developed an integrated model of health literacy. The model outlines four types of competencies (accessing, understanding, appraising, and applying health information) that are applied across multiple domains (health care, disease prevention, and health promotion). This framework is meant to guide health literacy scholarship and underscore the need for research and practice across a continuum of levels, in accordance with the socio-ecological model (McLeroy, Bibeau, Steckler, & Glanz, 1988), from the individual level to the population level (Sorensen et al., 2012). Based on this framework, and the call from the scientific community to increase interdisciplinary scholarship, the evidence base for health literacy research and practice could expand its scope by drawing from multiple disciplines.

To explore whether the published evidence base is reflective of and responsive to an integrated, interdisciplinary approach to health literacy, we examined trends in the health literacy literature over the past 25 years to answer the following research questions:

- Research Question 1 (RQ1): What disciplines contribute to the field of health literacy as measured by published literature?
- Research Question 2 (RQ2): How do disciplines draw from and contribute to an interdisciplinary evidence base as measured by citation networks?

## Methods

To provide a quantitative assessment of publication and citation patterns in the health literacy literature, we developed a keyword list to identify published journal articles over the past 25 years. The keyword list was developed through an iterative process and included health literacy and health information literacy, along with the most commonly used health literacy assessment tools. We decided to include the full name of the assessment tools as keywords (e.g., Rapid Estimate of Adult Literacy), as many articles that only used the abbreviations (e.g., REALM) were not relevant to the study. We employed the wildcard “*” to capture relevant variations of a word, and quotation marks were used to identify phrases. We decided to develop and use our own search query to limit our data set to articles specifically related to health literacy. The final query included the following:

- “*health literacy*” or “*test of functional health literacy*” or “*rapid estimate of adult literacy*” or “*rapid estimate of adolescent literacy*” or “*newest vital sign*” or “*health information literacy*” (title/abstract)
- “1991/01/01 – 2015/12/31” (date of publication)
- English [language]
- journal article (publication type) and review (publication type)

We conducted a search in four different bibliographic databases: Scopus, PubMed, CINAHL, and Web of Science. Journal and review articles were regarded as relevant if any of the terms were found in the title, abstract, or keyword fields. The query of bibliographic records between 1991 and 2015 resulted in over 4,000 records from each database: Scopus (5,442), PubMed (4,852), CINAHL (4,741), and Web of Science (4,063). Using the article title as the common variable for merging, data from the four databases were merged and duplicates were removed to produce 6,229 unique bibliographic records. For a quality check, 100 of the 6,229 articles were randomly selected and reviewed for study relevance, and all articles were relevant.

## Scientometric Analysis

We employed scientometric approaches to answer the research questions (Kim & Chen, 2015; Kim, Zhu, & Chen, 2016) and to build upon prior bibliometric analysis in health literacy research (Shapiro, 2010). CiteSpace is a scientometric suite of software developed by Chen (Chen, 2006), and has been used to generate and analyze networks of co-cited references using bibliographic records in various fields (Chen, Hu, Liu, & Tseng, 2012; Wei, Grubesic, & Bishop, 2015; Wu, Zhang, Hao, & Qin, 2016) and is part of a growing field of bibliometric research in the health sector (Bornmann & Leydesdorff, 2014; Buchan, Jurczyk, Isserlin, & Bader, 2016; Huang & Chang, 2012; Skvoretz et al., 2016). This tool computationally detects and renders thematic patterns and emerging trends in published science. CiteSpace also provides a visual representation called a dual-map overlay, which depicts domain-level citation paths (Chen & Leydesdorff, 2014); that is, we can visualize the path from the cited disciplines to the citing disciplines. Other similar software, including VOSviewer (Version 1.6.5; Centre for Science and Technology Studies, Leiden University [Nees Jan van Eck and Ludo Waltman], The Netherlands), Vantage Point (Version 10.0; Search Technology, Inc., Atlanta, GA), and HistCite (Clarivate Analytics [subsidiary of Thomson Reuters], Philadelphia, PA), support research profiling, constructing and visualizing networks of bibliographic units. However, CiteSpace is the only software to provide the unique functions of burst detection and dual-map overlay. These key functions provide a richer understanding of a field's trajectory by providing analyses that go beyond cumulative metrics to identify emerging themes and trends.

For RQ1, we used basic scientometric analysis to provide information on the disciplines that are contributing to the field of health literacy, including the number of published articles, cited journals, and subject categories. Each bibliographic database (e.g., Web of Science, PubMed), assigns subject categories to indexed journals, and articles published in these journals automatically inherit the journals' subject categories. To illuminate significant changes in publication trends, we used a burst detection algorithm to capture sharp increases in subject categories (Kleinberg, 2003). A subject category was regarded as “bursting” if it showed a significant increase in frequency during a specific duration of time.

For RQ2, we employed more advanced scientometric analysis, including network cluster analysis and citation path analysis, to better understand the interdisciplinary nature of published health literacy research. Network cluster analysis is a way to quantify how articles cite one another, with clusters forming around commonly and consistently cited article groups. Each node represents a cited article and the size of a node is proportional to its cited frequency. Nodes are grouped in the same lines by a clustering technique called smart local moving (Waltman & van Eck, 2013). Clusters are numbered in such a way that higher rankings are given to the clusters containing a higher number of references (1 being the highest ranking). For domain citation paths, a second type of scientometric analysis was used. Domains are journal-level features, and CiteSpace [Version 4.4.R1] creates domain names based on the top 3 terms in the journal name (and in some cases, conference names, when an article was presented at a conference proceeding). In the visualization, the left regions represent the journal-level domains where the retrieved articles publish (citing domain), whereas the right clusters indicate the journal-level domains from which they cite (cited domain) (Chen & Leydesdorff, 2014).

## Results

### RQ1: What Disciplines Contribute to the Field of Health Literacy as Measured by Published Literature?

**Figure 1.**
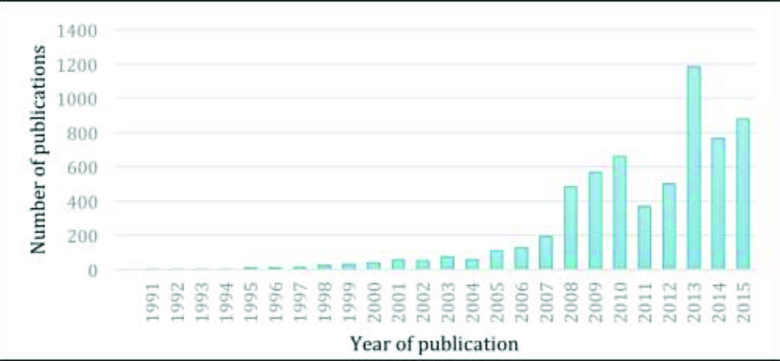
Yearly distribution of health literacy publications in the integrated data set (*n* = 6,229) (1991–2015).

**Figure 1** depicts the number of records over time in the final data set (*n* = 6,229). Health literacy has received increasing attention from the scientific community as measured by the published literature, with large spikes in 2008 and 2013.

**Table 1** describes the top 10 subject categories by frequency and density in our dataset. Density is defined as frequency divided by the number of years published (i.e., in our study, 2015 minus the “year first published” column). Frequency shows a snapshot of the cumulative impact of a subject category, whereas density provides an additional metric measuring the distribution of the impact over time. Among the subject categories that were first recorded in 1995, “public, environmental, and occupational health” received the most assignments (*n* = 1,110) and the largest density (52.9). In general, most subject categories relate to medical and clinical domains. The most recent addition in the top 10 list was “information science and library science,” first appearing in 2003 (*n* = 232). This subject category has a relatively high density given its first occurrence is the most recent among the leading categories, suggesting that recent health literacy literature has been largely driven by scientific approaches in information and library sciences.

**Figure 2.**
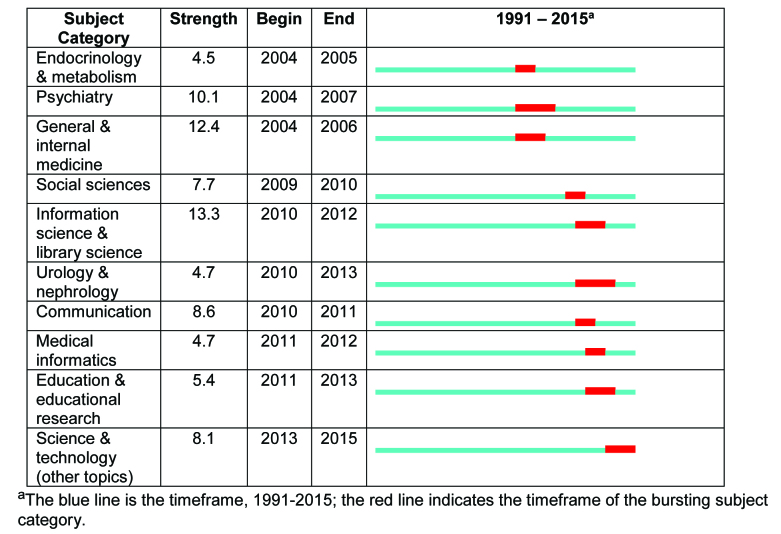
Top 10 bursting subject categories in health literacy publications (1991–2015).

**Figure 2** describes the top 10 “bursting” subject categories, sorted by the beginning year of the burst. Burst detection is a way to measure the intensity of subject categories during a specific timespan. In this study, social sciences and information sciences have received bursting attention in recent years. Findings from **Table 1** and **Figure 2** indicate that an early focus in health literacy research was on medical and clinical domains, with little overlap with social science, information science, and communication. Scholars from information and library sciences, communication, education, and science and technology appear to have developed an emerging focus on health literacy in recent years.

In addition to subject category, examining what journals have been the most frequently cited can serve as a proxy for what disciplines are contributing to the knowledge base in health literacy research. **Table 2** displays the top 10 most frequently cited journals in our dataset, the year it was first cited, and the density of how many times per year a specific journal has been cited, from its first year cited. Results show that two journals have been cited more than 2,000 times—the *Journal of General Internal Medicine* (*n* = 2,432) and *Patient Education and Counseling* (*n* = 2,252). These two journals were first cited early in the dataset, in 1995 and 1994, respectively, and they also demonstrate the greatest densities. The most recently cited journal to be added to the top 10 list is the *Journal of Health Communication* (*n* = 989), first cited in 2001. This journal also demonstrates a relatively high density (65.9), given its first year of citation was 2001. This finding suggests a recent concentration in publications in health literacy scholarship in the *Journal of Health Communication*.

### RQ2: How Do Disciplines Draw from and Contribute to an Interdisciplinary Evidence Base as Measured by Citation Networks?

**Figure 3.**
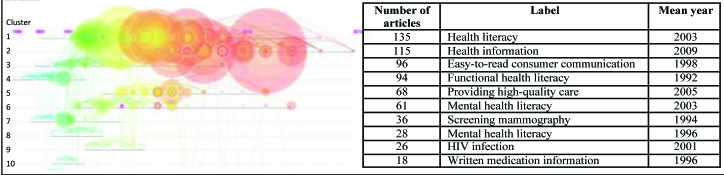
Health literacy co-citation network cluster timeline visualization (1991–2015).

**Figure 3** summarizes the 10 largest clusters in terms of the number of member articles, label extracted from titles and abstracts, and mean year of published citations. The color legend at the top indicates that citations in cooler colors occur closer to 1990 whereas hotter ones occur closer to 2015. Among these clusters, Cluster 4 is the oldest and the label generated from this line of research is functional health literacy, an earlier focus in the field. Considering both the size and recency of member nodes as depicted in the visualization, there are four emerging research themes reflected by Clusters 1, 2, 5, and 6: health literacy, health information, providing high-quality care, and mental health literacy.

**Figure 4.**
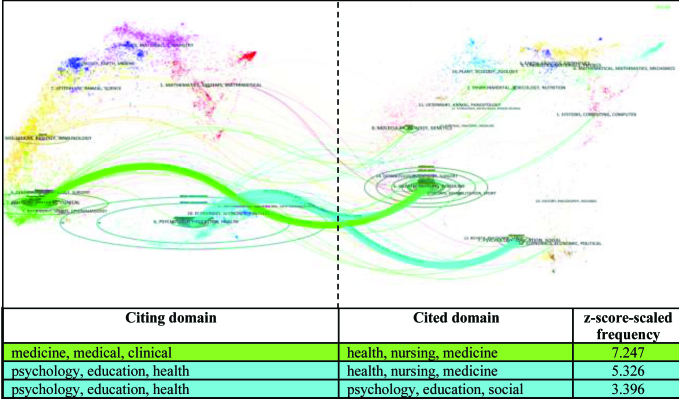
Domain-level citation trends in health literacy (1991–2015).

As depicted in **Figure 4**, there are three main citation paths in the health literacy data set. The base map depicts the interconnections of over 10,000 journals and these journals are clustered into regions that represent publications and citation activities at a domain level (Kim et al., 2016). Citation trajectories are distinguished by citing regions' colors. The thickness of these trajectories is proportional to the z-score–scaled frequency of citations; that is, the wider the path, the more frequently such citations occur. The relationships are sorted by the z-scores in descending order where the values are rounded to the nearest thousandth. Findings indicate that articles from the domains of “medicine, medical, clinical” heavily cite from one domain (health, nursing, medicine). This is displayed in the figure by the single green line connecting the two domains. On the other hand, health literacy articles from the domain “psychology, education, health” cite from two separate domains (health, nursing, medicine and psychology, education, social). This is displayed in **Figure 4** by the blue line drawing from two separate domains and converging on the one domain.

## Discussion

Despite the increased number of publications and the growing diversity of disciplines publishing in the field of health literacy, citation paths indicate the presence of a few, self-contained disciplines that contribute to the bulk of the literature, suggesting a lack of interdisciplinary research. However, new disciplines in health literacy research have emerged in recent years based on the examination of subject categories and cited journals that help to grow and diversify the knowledge base. It also suggests that there is potential to develop more interdisciplinary research in the future.

Our findings do not demonstrate that an integrated approach to health literacy as proposed by Sorensen et al. (2012) is occurring, per the lack of interdisciplinary citation networks. Moreover, although there is some support for movement and momentum toward an integrated framework, as demonstrated by recent additions of fields outside of the traditional health sector, more must be done to incorporate multiple dimensions of the health environment continuum, including both individual- and population-level domains. However, the relatively recent re-focusing of an integrated approach to health literacy research (circa 2008) may take time to be reflected in the published literature.

Our findings contribute to the health literacy field by providing empirical, bibliometric data that describe how various disciplines are drawing from and contributing to the published literature. The integrated health literacy model was an effort to conceptualize and contextualize various components of health literacy scholarship, laying out a much-needed framework from which to develop measures and test multiple levels of intervention. If there is an interest in the field to move toward an integrated approach, involving multiple domains from the individual to population levels, our findings suggest that more can be done to conduct integrated work and ensure it is translated to the published literature.

An important evolution of health literacy research, as observed in the co-citation cluster analysis, is the shifting focus away from specific diseases to more general applications. This is evident as cluster labels from earlier health literacy literature were more specific applications of health literacy, including screening mammography, HIV infection, and written medical information, whereas more recent cluster labels include health literacy and health information. These findings further support the evolution of an integrated approach to health literacy, as recent models and frameworks of health literacy underscore competencies and domains that transcend disease- or behavior-specific applications. As the field continues to adopt a more integrated approach to interdisciplinary research, the clusters of health literacy and health information are likely to continue to expand upstream, and may soon include terms such as health disparities and social determinants. At the same time, it is important to recognize the value of disease- and condition-specific research, and such research should continue to identify key issues and potential interventions.

Of the two leading domains of disciplines that publish health literacy research, one heavily cites from one domain, whereas the other heavily cites from two domains. Medical and clinical research may rely more on one line of research, that is, other clinical research, because of similarities in patient populations or the clinical context in which research is conducted, and thus will cite more heavily from the same domain. Although clinical research on its own is important and should continue to gather evidence and create knowledge in the field of health literacy, adding more integrated research may also be beneficial. For instance, health literacy in the health care domain is likely to be impacted and influenced by the domains of disease prevention and health promotion, suggesting an opportunity to engage and work with disciplines outside of the health care setting.

## Limitations

There are several limitations worth noting. First, the software used in this study, CiteSpace, only accepts Web of Science-formatted records. Records retrieved from other databases go through a conversion process implemented in the software. For this study, less than 5% of records retrieved from other databases were not able to be converted, leading to a small percentage of data loss. Despite this, integrating data across PubMed, Scopus, and CINAHL with Web of Science produced 6,229 unique articles and supports our goal to explore more comprehensive published literature. In addition, CiteSpace captures intellectual trajectories (i.e., domains and labels) from collective clusters of articles, as opposed to article-level citation paths, limiting our ability to analyze trajectories of landmark articles in health literacy. For this study, however, focusing on domain-level trajectories allowed for commentary on the larger field of health literacy, and not on individual articles. Second, our search term list may not have captured the full population of health literacy articles relevant to our study. To minimize this bias, we used an iterative process to develop the keyword list and examined additional articles that were included based on additional search term queries (e.g., literacy and health outcomes). We determined that the additional articles were not relevant to our study (e.g., they only had a literacy focus). Finally, we are operating under the assumption that subject categories, journal titles, domains, and citation networks are valid and reliable measures used to capture the true nature of discipline-specific health literacy scholarship. We believe that this is an accurate measure of discipline activity as there is a rich history and tradition of discipline-specific scholarship being published and disseminated in discipline-specific journals.

## Conclusion

This study is one of few initial assessments of citations in health literacy research. To provide additional insight into differences observed in health literacy citation paths, and to further understand the nature of interdisciplinary research, future work may wish to examine and compare the composition of study teams and authors. For example, research teams publishing in the medical domain may be composed of clinical researchers and clinicians, whereas teams publishing in the education domain may be composed of more diverse disciplines, including clinicians as well as disciplines outside of clinical research, thus leading to more citation paths. Future work may also want to expand the search terms, or look at specific populations (such as adolescents) or topics (such as diabetes) to make recommendations for future research directions.

Finally, for scholars in the field who hope to increase interdisciplinary research, our findings support the need for more deliberate and strategic activities to help strengthen opportunities for interdisciplinary health literacy work (Allen, Auld, Logan, Montes, & Rosen, 2017). Developing opportunities and resources for researchers from diverse and complementary fields to work together would strengthen a team science approach, and would likely lead to innovative approaches to the study of health literacy and design of interventions.

## Figures and Tables

**Table 1:** Top 10 Subject Categories in Health Literacy Publications (1991–2015)

**Subject Category**	**Frequency**	**Year of First Appearance**	**Density^a^**
Public, environmental, and occupational health	1,110	1995	52.9
Health care sciences and services	626	1995	29.8
General and internal medicine	465	1995	22.4
Medicine	427	1995	20.3
Psychiatry	387	1997	20.4
Nursing	319	1998	17.7
Psychology	299	1996	15
Health policy and services	289	1998	16.1
Social sciences (other topics)	241	1996	12.1
Information science and library science	232	2003	17.8

a Frequency of subject category assignments divided by the duration between “year” and 2015.

**Table 2:** Top 10 Cited Health Literacy Journals (1991–2015)

**Cited Journals**	**Frequency**	**Year First Cited**	**Density^a^**
*Journal of General Internal Medicine*	2,432	1995	115.8
*Patient Education and Counseling*	2,252	1994	102.4
*Journal of the American Medical Association*	1,918	1995	91.3
*Archives in Internal Medicine*	1,552	1996	77.6
*Annals in Internal Medicine*	1,299	1998	72.2
*American Journal of Public Health*	1,282	1995	61
*Social Science and Medicine*	1,280	1999	75.3
*Family Medicine*	1,161	1993	50.5
*Medical Care*	1,113	1998	61.8
*Journal of Health Communication*	989	2001	65.9

a Frequency of citations divided by the duration between “year” and 2015.
